# Correlation between antioxidant effect mechanisms and polyphenol content of *Rosa canina*

**DOI:** 10.4103/0973-1296.66943

**Published:** 2010

**Authors:** Hasan Kılıçgün, Dehen Altıner

**Affiliations:** *Department of Nutrition and Dietetic School of Health, University of Erzincan, 24100, Erzincan, Turkey*; 1 *Department of Biochemistry, Faculty of Pharmacy, Marmara University, Haydarpaşa 81010, Istanbul, Turkey*

**Keywords:** Antioxidant, phenolics, prooxidant, *Rosa canina*

## Abstract

*Rosa canina* L. is a member of Rosaceae family, which is well-known for its high phenolic contents. These compounds are known to possess antioxidant, antimutagenic and anticarcinogenic effects. However, they have yet to pass controlled clinical trials for efficacy, and their potential for prooxidant activity is an understudied field of research. In order to estimate the correlations between phenolic contents and antioxidant/proxidant effect mechanisms, different concentrations of *R. canina* fruit extracts were examined in this study. *R. canina* showed antioxidant activities at all concentrations with respect to the reducing power, hydrogen peroxide scavenging activity and superoxide anion radical scavenging (O2^ः-^) activity assays, whereas a negative correlation was observed with the metal ion chelating activity and free radical scavenging activity [1,1-diphenyl-2-picryl-hydrazil (DPPH) % inhibition] assays at higher concentrations with the phenolic content of *R. canina*. These results suggest that *R. canina* may act not only as an antioxidant, but also as a prooxidant with the effects depending on its concentrations.

## INTRODUCTION

In the past few years, polyphenols have gained much more attention and have become an important focus of research interest, owing to their antioxidant activities and various beneficial effects on human health, such as antioxidant, antimutagenic and anticargionogenic effects, as well as their ability to modify gene expression.[1,2] However, it remains unclear whether this protective effect is attributable to the phenols or to other agents in the diet. On the other hand, it has been shown that some plant phenolics can have prooxidant activities under certain conditions.[3,4] Members of the Rosaceae family have long been used for food and medicinal purposes. Their fruits are rich in polyphenols. The physiological functions of Rosaceae fruits may be partly attributed to the abundance of phenolics in them. *Rosa canina* L. is a member of Rosaceae family. Because of its significant nutritional and therapeutic benefits, it has been used both traditionally and for medicinal purposes.[5,6] There are some studies on the antioxidant properties of R. *canina*.[7–9] As far as we know in the best way, there have not been studies reporting the prooxidant activity of R. *canina.* Therefore, the objectives of this study were to evaluate the concentration-dependent potential antioxidant and prooxidant effects of wild R. *canina*.

## MATERIALS AND METHODS

All chemicals were purchased from Merck, Fluka Chemika and Sigma In order to use the fruits of R. *canina* in this study, they were collected from Erzincan, Turkey. After the fruits were dried, seeds were removed from the collected plant material. Infusion was made by pouring 100 ml of boiling water on 8 g of plant material. The mixture was left to stand for 20 min and then filtered and diluted to the concentrations (1, 2, 3, 4, 8% g/100 ml).

Total phenol content was determined by the method adapted from Vinson,[10] using the Folin–Ciocalteu reagent.[11] To each tube, 1.5 ml of R. *canina* extract, 1 ml of HCl and 5 ml of methanol were added. The tubes were capped, mixed thoroughly and heated at 90°C for 2 hours. After 20 min, the solution was made up to 10 ml with distilled water. The solution was filtered and Folin-Ciocalteu reagent was added to the solution. The tubes were capped, mixed thoroughly and the blue coloration was read at 750 nm against a blank standard. Results were expressed in milligrams of catechin/l of plant material infusion.

Reducing power of R. *canina* concentrations was measured using the method of Oyaizu.[12] Different concentrations of R. *canina* (1, 2, 3, 4, 8% g/100 ml) in 1 ml were mixed with phosphate buffer (2.5 ml, 0.2 M, pH 6.6) and potassium ferricyanide [K_3_Fe-(CN)_6_] (2.5 ml, 1%). The mixture was incubated at 50°C for 20 min. A portion (2.5 ml) of trichloroacetic acid (10%) was added to the mixture. Then, it was centrifuged for 10 min at 3000 rpm. The upper layer of solution (2.5 ml) was mixed with distilled water (2.5 ml) and FeCl_3_ (0.5 ml, 0.1%), and the absorbance was measured at 700 nm using a spectrophotometer (Beckman DU 520).

H_2_O_2_ scavenging activity of R. *canina* concentrations was measured by the method of Ruch and colleagues.[13] A solution of hydrogen peroxide (40 mM) was prepared in phosphate buffer (pH 7.4). Hydrogen peroxide concentration was determined spectrophotometrically by measuring the absorption at 230 nm using a spectrophotometer (Beckman DU 520). The concentrations of R. *canina* (1, 2, 3, 4, 8 g/100 ml) were added to a hydrogen peroxide solution (4 ml R. *canina* infusion + 0.6 ml H _2_O_2_ ). Absorbance of mixtures at 230 nm was determined after 10 min against a blank solution containing phosphate buffer without hydrogen peroxide.

Superoxide anion radical scavenging activity (O2^·–^ % inhibition) was detected by the reduction of nitroblue tetrazolium (NBT), essentially as described by Nishikimi *et al*.[14] A typical assay mixture is made up of 3 mM hypoxanthine solution, 100 ml/U xanthine oxidase solution, 12 mM diethylene triamine pentoacetic acid (DETAPAC) solution and 178 mM NBT solution. All the solutions were prepared in 0.1 M potassium phosphate buffer (pH 7.4). The mixture contained 1 ml hypoxanthine + 1 ml xanthine oxidase + 1 ml DETAPAC + 1 ml NBT + 1 ml R. *canina* infusion in a total volume of 5.0 ml. After mixing, absorbance was recorded at 560 nm against a blank, which did not contain the compound, at different time intervals.

Free radical scavenging activity[1,1-diphenyl-2-picryl-hydrazil (DPPH) % inhibition] was determined by the method of Yamaguchi and colleagues.[15] Exactly 500 µM solution of DPPH in absolute ethanol was prepared. Then, 1 ml of the solution was added to 0.2 ml of R. *canina* solution at different concentrations (1, 2, 3, 4, 8% g/100 ml). Also, 0.8 ml of Tris-HCl was taken and 100 mM Tris-HCl buffer (pH 7.4) and added to the solution. The mixture was shaken vigorously and allowed to stand in darkness and at room temperature for 20 min. Then the absorbance was measured at 517 nm using a spectrophotometer (Beckman DU 520).

Metal ion chelating activity of R. *canina* concentrations was determined by the method of Decker and Welch.[16] The concentrations of R. *canina* (1 ml) (1, 2, 3, 4, 8% g/100 ml) were added to a solution of 2 mM FeCl_2_ (0.1 ml). The reaction was initiated by the addition of 5 mM ferrozine (0.2 ml) and the mixture was shaken vigorously and left to stand at room temperature for 10 min. After the mixture had reached equilibrium, the absorbance of the solution was measured spectrophotometrically at 562 nm using a spectrophotometer (Beckman DU 520).

### Statistics

Data are presented as mean ± SD of at least three independent experiments (*n* = 3). One-way analysis of variance (ANOVA) followed by Scheffe‘s test were performed to determine statistical differences between groups with the aid of SPSS software version 11.0 (SPSS, Chicago, IL, USA). Statistical significance was defined as *P* < 0.05 for all tests.

## RESULTS AND DISCUSSION

Plant polyphenols are aromatic hydroxylated compounds, commonly found in vegetables, fruits and many food sources. Polyphenolic compounds are potential antioxidant substances and protective agents against the development of human disease. On the other hand, there are also reports like that of Goldman *et al*.[17] describing the prooxidative properties of phenolic compounds. In this study, the antioxidant and prooxidant activities of R. *canina* fruits‘ infusions were evaluated according to a direct interaction between the reducing power, H2O2 scavenging activity, O_2_^·–^ scavenging activity, the metal ion chelating activity, DPPH % inhibition and total phenol concentrations. In order to determine the antioxidant/prooxidant activities of R. *canina*, we used five different antioxidant assays in this study. Due to oxidative processes, the total antioxidant activities of an antioxdants cannot be evaluated by using one single method. Therefore, at least two methods should be performed in order to evaluate the total antioxidant/prooxidant activities.

A number of studies showed that antioxidant activity of plant extracts is correlated with total phenolics rather than with any individual phenolic compound.[18–20] So, total phenol content of R. *canina* was investigated in this study. Notable correlation-dependent concentration was observed between total phenol content and concentrations of R. *canina*. The total phenol content of R. *canina* concentrations is presented in Table 1.

**Table 1:** Total phenol content of *R. canina* infusions

Parameters	1%	2%	3%	4%	8%
Total phenol content (mg/lcatechin equivalent)	425.0 ± 2.65a	815.7 ± 3.52b	1023.3 ± 3.05c	1472 ± 2.65d	2124.3 ± 3.52e

Means ± SD, *n* = 3, Different letters on the same line are considered to be statistically significant when *P* < 0.05

In the assay for reducing power, R. *canina* exhibited a high antioxidant capacity at all concentrations. Reducing power of R. *canina* fruits is presented in Table 2. Higher absorbance of the reaction mixture indicated greater reducing power. Significant concentration-dependent correlation was observed between reducing power and total phenol content of R. *canina*. These results suggest that R. *canina* has an antioxidant effect.

**Table 2:** Effect of concentrations of *R. canina* infusions on antioxidant mecanisms

Parameters	Control	1%	2%	3%	4%	8%
Reducing power (A_700_)	0.005 ± 0.0004a	0.216 ± 0.0051b	0.235 ± 0.0031c	0.248 ± 0.0058c	0.261 ± 0.0031d	0.292 ± 0.0040e
H_2_O_2_ scavenging activity (mM H_2_O_2_)	4.867 ± 0.2082a	0.141 ± 0.0031b	0.114 ± 0.0015b	0.104 ± 0.0060b	0.024 ± 0.0020b	0.041 ± 0.0427b
O_2_ ·– superoxide radical scavenging activity (% inhibition)	0.00	50.170 ± 0.765a	64.903 ± 0.556b	72.540 ± 0. 433c	76.237 ± 0.666d	84.453 ± 0.586e
Free radical scavenging activity (% DPPH inhibition)	0.00	84.043 ± 0.340a	87.970 ± 0.135b	96.250 ± 0.036c	88.040 ± 0.199b	67.193 ± 0.986d
Metal ion chelating activity (A_562_)	0.493 ± 0.0010a	0.421 ± 0.0015b	0.379 ± 0.0029c	0.343 ± 0.0025d	0.315 ± 0.0042e	0.326 ± 0.0015f

Means ± SD, *n* = 3, Different letters on the same line are considered to be statistically significant when *P* < 0.05

The *in vitro* estimation of the effect of the R. *canina* infusions on H_2_O_2_ showed their activity to scavenge this substance[Table 2] in a concentration-dependent manner. Significant correlation was observed between H_2_O_2_ scavenging activity and the total phenol content of R. *canina*. As a matter of fact, Daels *et al*. recently demonstrated that the *in vivo* and *ex vivo* inhibitory effects of R. *canina* against H_2_O_2_ were in a dose-dependent manner.[7]

When the *in vitro* superoxide anion radical scavenging potential of R. *canina* infusions was tested, R. *canina* infusions provided a strong scavenging effect against superoxide radical. But the antioxidative activity of the individual concentrations differed significantly [Table 2]. The results indicate that R. *canina* exerts its therapeutic effect for treating radical-related pathological damage by scavenging the free radicals. On the other hand, Daels *et al*. recently demonstrated that R. *canina* exhibited *in vivo* and *ex vivo* inhibitory effects against superoxide anion in a dose-dependent (0.5–50 mg/l) manner.[7]

The scavenging activity on DPPH radical was related to the concentration of R. *canina* infusions; the activity increased significantly as a result of increasing concentration (1, 2 and 3%). But, DPPH radical scavenging activity did not show an increasing trend at higher concentrations (4 and 8%). Moreover, the scavenging activity dramatically decreased at higher concentrations [Table 2]. This information suggests that the same plant that optimizes antioxidant capacity may also act as a prooxidant in different test systems, depending on its concentration.

The potential metal ion chelating activity of R. *canina* infusions was tested at different concentrations (1–8% g/100 ml). It was seen that lower concentrations (1, 2, 3 and 4%) of R. *canina* indicated higher metal ion chelating activity. But the same chealating activity was not seen at higher concentration (8%) of R. *canina*. Furthermore, R. *canina* showed a decreasing chelating trend at a concentration of 8% [Table 2]. This effect may be attiributed to polyphenol compounds of R. *canina*.[21,22]

Finally, the correlations obtained quantitatively confirm the parallelism between the polyphenol amount and the reducing power, H_2_O_2_ scavenging activity and O_2_^·–^ scavenging activity assays. But the same parallelism between the polyphenol amount and metal ion chelating activity and DPPH % inhibition assays was not seen at the higher concentrations of R. *canina* infusions. Because of these considerations, the data definitely demonstrate that the dual effect of R. *canina* depends on the concentration. These results suggest that R. canina may act not only as an antioxidant, but also as a prooxidant, with the effects depending on its concentrations. This study can be useful for the sustainable future use of this plant. On the other hand, further studies should be continued to get proper information regarding the role of R. *canina* as a prooxidant and its involvement in the other dose-dependent processes.
